# A Mycobacterial Enzyme Essential for Cell Division Synergizes with Resuscitation-Promoting Factor

**DOI:** 10.1371/journal.ppat.1000001

**Published:** 2008-02-29

**Authors:** Erik C. Hett, Michael C. Chao, Lynn L. Deng, Eric J. Rubin

**Affiliations:** 1 Department of Immunology and Infectious Diseases, Harvard School of Public Health, Boston, Massachusetts, United States of America; 2 Department of Medicine, VA Medical Center, Boston, Massachusetts, United States of America; Johns Hopkins School of Medicine, United States of America

## Abstract

The final stage of bacterial cell division requires the activity of one or more enzymes capable of degrading the layers of peptidoglycan connecting two recently developed daughter cells. Although this is a key step in cell division and is required by all peptidoglycan-containing bacteria, little is known about how these potentially lethal enzymes are regulated. It is likely that regulation is mediated, at least partly, through protein–protein interactions. Two lytic transglycosylases of mycobacteria, known as resuscitation-promoting factor B and E (RpfB and RpfE), have previously been shown to interact with the peptidoglycan-hydrolyzing endopeptidase, Rpf-interacting protein A (RipA). These proteins may form a complex at the septum of dividing bacteria. To investigate the function of this potential complex, we generated depletion strains in *M. smegmatis*. Here we show that, while depletion of *rpfB* has no effect on viability or morphology, *ripA* depletion results in a marked decrease in growth and formation of long, branched chains. These growth and morphological defects could be functionally complemented by the *M. tuberculosis ripA* orthologue (*rv1477*), but not by another *ripA-*like orthologue (*rv1478*). Depletion of *ripA* also resulted in increased susceptibility to the cell wall–targeting β-lactams. Furthermore, we demonstrate that RipA has hydrolytic activity towards several cell wall substrates and synergizes with RpfB. These data reveal the unusual essentiality of a peptidoglycan hydrolase and suggest a novel protein–protein interaction as one way of regulating its activity.

## Author Summary

Mycobacteria, like all peptidoglycan-containing bacteria, must extend and cleave the surrounding structurally rigid layer of peptidoglycan to grow and divide. The peptidoglycan hydrolases responsible for this cleavage often have redundant functions, both revealing their importance and making them difficult to study. Furthermore, such hydrolases must be tightly regulated, due to their potentially lytic nature. We recently demonstrated the interaction between a lytic transglycosylase (Rpf) and an endopeptidase (RipA) at the septum of dividing bacteria. To investigate the role of these two hydrolases, we generated conditional mutants of each and were surprised to find that depletion of *ripA* resulted in long chains of cells. This phenotype was reversed upon induction of *ripA*, indicating that cell wall expansion and septum formation can be decoupled from the process of septum resolution. In addition, we present data showing that the combination of Rpf and RipA results in enhanced hydrolysis of peptidoglycan in an in vitro assay, suggesting protein–protein interactions as one potential mechanism of regulation.

## Introduction

Though not formally considered virulence factors, genes required for bacterial cell division clearly are necessary for the growth, and thus, pathogenesis, of bacteria. The distinction between homeostatic and virulence genes is blurred when nonessential genes involved in vegetative cell division become essential under specific stressful conditions encountered inside a host. Such an example is seen with the resuscitation-promoting factors (Rpf) encoded by many different bacteria, including mycobacteria. These proteins are named for their ability to resuscitate nonreplicating dormant bacteria. The single Rpf-encoding gene in *Micrococcus luteus* [1] is essential, but as many as three of the five genes encoding RpfA-E can be deleted in *Mycobacterium tuberculosis* without markedly affecting *in vitro* growth. However, one single deletion (*rpfB*) and several of the triple combinations yielded strains unable to grow or divide in stressful conditions in vitro and in vivo [2],[3]. This suggests that certain potential cell division proteins that appear to play nonessential roles in homeostatic processes can become vital in conditions of stress.

The vital processes of cell growth and division involve the temporal and spatial coordination of events such as peptidoglycan and cell wall extension, DNA replication, chromosomal partitioning, Z-ring assembly, septum formation, and cytokinesis. Much of the mechanism behind this coordination involves inhibiting and stabilizing proteins that regulate the eventual assembly of a Z-ring at the midcell of bacteria [4],[5]. This Z-ring consists primarily of a polymerized ring of tubulin-like FtsZ on the cytoplasmic side of the plasma membrane, stabilized by membrane-associated and integral membrane proteins. Assembly occurs in an ordered fashion that is not entirely linear, with some components assembling before joining the Z-ring [6],[7],[8]. Some of the last proteins to be recruited to the Z-ring are thought to be the peptidoglycan hydrolyzing enzymes, such as AmiC [9] and EnvC [10] in *E. coli*. These enzymes digest the peptidoglycan layers connecting two recently developed daughter cells in the final stage of cell division [11]. While crucial to cell division, the regulation of these potentially lethal enzymes is poorly understood. It is thought that protein-protein interactions play a role in regulating activity and localization [12]. Studying cell wall hydrolases has proven difficult due to the large number encoded in most bacterial genomes and the high degree of functional redundancy. For example, seven hydrolases can be deleted from a strain of *E. coli* without loss of viability [13].

Little is known about the hydrolases involved in mycobacterial cell division. CwlM and Rv2719c were both shown to be mycobacterial cell wall hydrolases [14],[15]. Two recently identified hydrolases, RpfB and RpfE, were shown to interact with rpf-interacting protein (RipA), a peptidoglycan endopeptidase [16]. Rpf proteins constitute a family of lytic transglycosylase enzymes capable of hydrolyzing the glycosidic bonds in the essential stress-bearing, shape-maintaining peptidoglycan layer [17]. RpfB has a structure similar to the *E. coli* soluble lytic transglycosylase 70 (Slt70) [18] and is known to hydrolyze the beta-1,4-glycosidic bond between N-acetyl muramic acid and N- acetyl glucosamine [19]. RipA has been shown to be a peptidoglycan hydrolase [16]. It is predicted to function as an L,D-endopeptidase, capable of hydrolyzing D-glutamyl-meso-diaminopimelic acid [20], two amino acids that are part of the crosslinking peptides vital for keeping peptidoglycan rigid and stable [21]. Both RpfB and RipA localize to the septa of dividing bacteria [16] and thus may play a role in the late stages of mycobacterial cell division, possibly during regrowth from a stressed state.

Here we show that depletion of *ripA* in a strain of *M. smegmatis* results in a significant decrease in growth, formation of long, branched chains, and increased sensitivity to a cell wall–targeting antibiotic. These defects can be functionally complemented with the *M. tuberculosis* allele of *ripA*. We demonstrate that the peptidoglycan hydrolytic activity of RipA synergizes with RpfB. Thus, this protein is an unusual example of an essential peptidoglycan hydrolase whose activity may be partially regulated through protein-protein interactions.

## Results

### Depletion of RipA blocks normal cell division

RipA interacts with RpfB, a lytic transglycosylase, and colocalizes at the septum [16]. We hypothesized that the RipA-RpfB complex may be involved in degrading peptidoglycan at the septum during cell division. To further investigate the function the individual components of this complex, we attempted to make deletion strains of *ripA* and *rpfB* in *M. smegmatis*. Though disruption of *rpfB* was successful, we were unable to disrupt the *ripA* gene in *M. smegmatis*. We have previously reported that the *ripA* gene in *M. tuberculosis* appears to be essential for in vitro growth [22], suggesting that it might also be essential in *M. smegmatis*.

**Figure 1:**
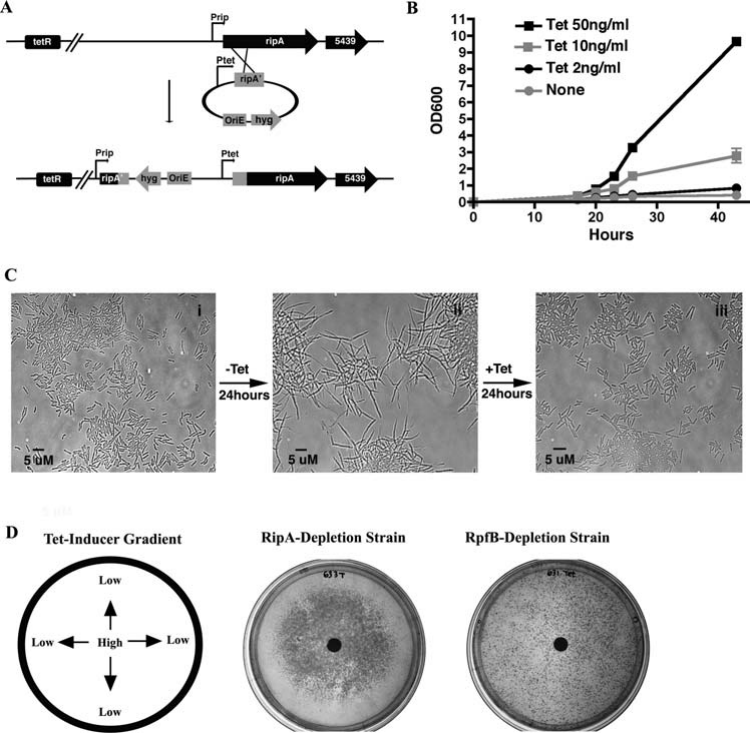
Depletion of RipA results in a reversible arrest in growth of *M. smegmatis.* (A) Diagram showing the strategy used to replace the native promoter of the *ripA-B* operon (Prip) with a tetracycline-inducible promoter (Ptet) through homologous recombination (strategy and diagram adapted from [28]). OriE: *E. coli* origin of replication. (B) *ripA* depletion strain of *M. smegmatis* was grown with inducer (Tet), then inoculated into media with decreasing amounts of inducer and followed by OD_600_ over time. Cultures with none or 2 ng/ml inducer grew in tight clumps that resulted in underestimation by OD_600_. Data are represented as mean+/−SEM. (C) Series of DIC micrographs of the *ripA* depletion strain of *M. smegmatis*. Depicts bacteria first grown with 50 ng/ml tetracycline inducer (i), then transferred to media lacking inducer for 24 hours (ii), and finally transferred to media with inducer for 24 hours (iii). Bacteria were visualized with a 100× objective. (D) Depletion strains of *M. smegmatis* grown on plates containing a gradient of inducer created by placing 10 µl of 10 ng/ml Tet on a paper disc in the center of the plate, resulting in a concentration of inducer highest at the middle of the plate and lowest at the edges. Colonies formed in a Tet-dependent manner for the RipA-depletion strain, while colonies from the RpfB-depletion strain grew independent of inducer.

To test this possibility we constructed depletion strains of *M. smegmatis* in which *ripA* (*MSMEG3153*) or *rpfB* (*MSMEG5439*) are transcribed from an inducible tetracycline promoter (Ptet, Figure 1A). We found that the *ripA* depletion strain had dramatically reduced growth in media lacking inducer (Figure 1B), while the *rpfB* depletion strain had normal growth in the absence of inducer. Both *ripA* and *rpfB* depletion strains grew normally in the presence of inducer. The growth phenotype seen with *ripA* depletion, as measured by optical density, was dose-dependent. While the optical density of *ripA* depleted cultures failed to increase in the absence of inducer, we did observe that bacteria formed visible clumps that increased in size during incubation. These clumps (due to filamentation) failed to suspend well and, therefore, were poorly measured using spectrophotometry. Remarkably, this phenotype is reversible, as addition of inducer to growth-arrested, *ripA* depleted cells resulted in the resumption of normal growth (Figure 1C). This indicates that the frequency of septum resolution can be uncoupled from septum formation and cell elongation. High levels of inducer did not result in gross morphological changes or lysis.

To further confirm the requirement of *ripA* for growth, we plated *ripA*-depletion or *rpfB*-depletion strains of *M. smegmatis* on plates containing a gradient of inducer. The *ripA*-depletion strain grew in a Tet-dependent manner, with the highest growth in the center corresponding to the highest concentration of inducer and no growth near the edges of the plate where inducer was the lowest, confirming the requirement of *ripA* for growth. Conversely, the *rpfB*-depletion strain grew similarly throughout the plate in a Tet-independent manner, indicating that *rpfB* is not required for growth under these conditions.

### Depletion of RipA results in abnormal morphology

**Figure 2:**
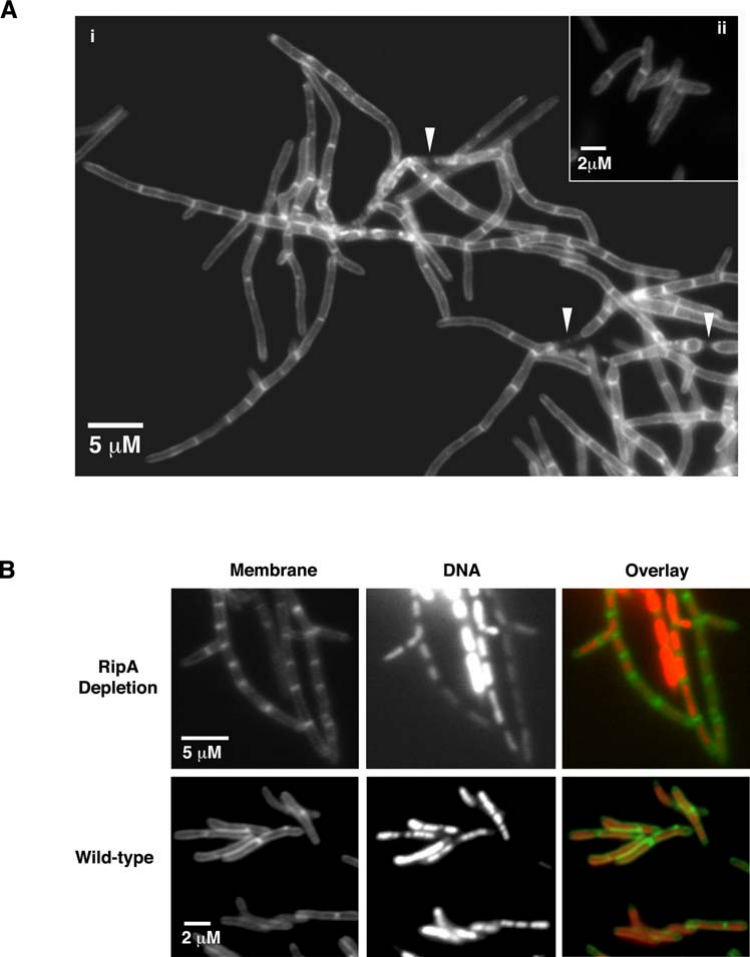
Depletion of RipA results in branching and chaining of *M. smegmatis*. (A) Micrographs of *M. smegmatis* strains with membranes imaged by staining with TMA-DPH. i. *ripA* depletion strain chains and branches when depleted of *ripA* for 24 hours (no inducer), ii. Wild-type. Arrowheads indicate regions where the cell wall appears pinched or lysed. Bacteria were visualized with 100× objective. (B) Micrograph of a branch of *ripA* depleted *M. smegmatis* or wild-type strains. Membranes (green) and DNA (red) were stained with TMA-DPH or SYTO 9, respectively, revealing apparent functional septation and DNA segregation. Bacteria were visualized with 100× objective.

While *rpfB* depleted cells had normal morphology, *ripA* depleted cells had markedly abnormal shape (Figures 1C and 2A). They grew in long, branched chains that account for the clumps seen grossly. Staining with the fluorescent membrane dye, TMA-DPH, revealed periodic septa along the chains of bacteria. DNA staining with SYTO 9 revealed nucleoids along the length of the chained bacteria, separated by septa, indicating that DNA segregation and septum formation processes are intact (Figure 2B). Occasional patches where the cell wall appeared pinched or partially degraded were also observed. It is not clear if these represent defective division sites or locations where bacteria had begun to lyse.

Branches, not observed in wild-type cells, were seen in almost all r*ipA*-depleted bacteria visualized (>95%). Interestingly, 91% (203/223) of the branches visualized originated directly adjacent to septa.

### *M. tuberculosis ripA* allele functionally complements *M. smegmatis ripA*

**Figure 3:**
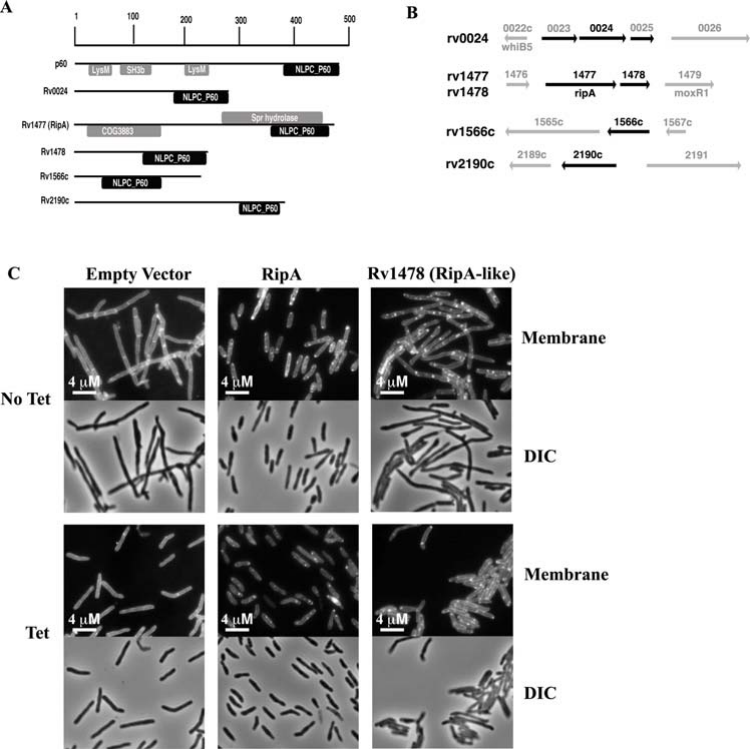
*M. tuberculosis ripA* allele complements depletion. (A) Predicted domains of *M. tuberculosis* RipA-like proteins and *L. monocytogenes* p60. The NLPC_P60 putative domain in p60 was previously shown to have endopeptidase activity against cell wall material and defines the family. (B) Gene neighbors and predicted operons of *rip* genes in *M. tuberculosis*. Operons are indicated with black arrows, *rip* genes are in black text, gene numbers are given above arrows and further annotation for some genes is provided below arrows. One gene on either side of the *rip*-operon is shown with gray arrows. (C) A *ripA-smeg* depletion strain containing the *M. tuberculosis ripA* allele, *ripA-mtb,* on an episomal construct with its native promoter grew like wildtype when depleted of *ripA-smeg* (no tetracycline), while a strain carrying an empty plasmid formed chains when *ripA-smeg* was depleted. The *M. tuberculosis* allele of the *ripA* paralogue, *rv1478*, was not able to complement *M. smegmatis* depleted of *ripA*. All strains grew like wildtype in the presence of inducer, though the RipA and RipA-like strains grew slightly shorter than empty vector in the presence of Tet. Membranes were visualized with TMA-DPH. Bacteria were visualized with 100× objective.

The *M. tuberculosis ripA* gene encodes a 472 amino acid protein that has been shown to degrade peptidoglycan [23]. Its C-terminal 105 amino acids contain a putative endopeptidase domain, which has 40% identity with the *Listeria monocytogenes* p60 protein (Figure 3A). p60 has been shown to be a cell wall endopeptidase by its ability to degrade cell wall [24],[25]. *ripA* is the first gene in a bicistronic operon. *rv1478*, the downstream gene, encodes a 241-amino acid protein, consisting of a signal sequence followed by a sequence homologous to the C-terminal half of RipA (70% putative hydrolase domain identity and 27% overall amino acid identity). Both genes in the apparent *ripA* operon encode predicted endopeptidase domains similar to a known p60 hydrolase [24] (Figure 3A). Because the inserted tetracycline-inducible promoter lies upstream of the operon, presumably transcription of both is dependent on the presence of inducer. Thus, either gene could be responsible for the observed phenotype. There are at least five *p60-*like genes in many of the mycobacterial species, including *M. tuberculosis*, *M. smegmatis*, and *M. bovis* BCG. These include *rv0024*, *rv1477* (*ripA*), *rv1478*, *rv1566c*, and *rv2190c*. These genes lie in various genomic contexts and there is no function discernable from genomic synteny (Figure 3B).

To identify the responsible gene we tested if the *M. tuberculosis* allele of *ripA* (*ripA-mtb*) was able to complement the *M. smegmatis* with diminished native *ripA* (*ripA-smeg*) production. We expressed *ripA-mtb* from a zeocin-marked episomal plasmid. A *ripA-smeg* depletion strain containing the *ripA-mtb* construct grew similarly to wildtype in the absence of inducer, while a strain carrying an empty plasmid formed chains when *ripA-smeg* was depleted (Figure 3C). In contrast, the *M. tuberculosis* allele of the *ripA* paralogue, *rv1478*, was not able to complement an *M. smegmatis ripA* depletion. These results confirm that *ripA* is sufficient for complementing the strain depleted of the *ripA* (*ripA-MSMEG3154*) operon and show that *ripA-mtb* is functionally similar to *ripA-smeg*.

### Depletion of RipA increases susceptibility to a cell wall–targeting antibiotic

**Figure 4:**
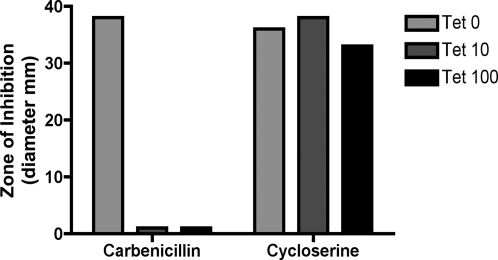
An *M. smegmatis* strain depleted of RipA is sensitive to a cell wall–targeting antibiotic. The *ripA* depletion strain of *M. smegmatis* was spread on LB agar plates containing different amounts of anhydrotetracycline inducer (ng/ml concentrations) to regulate the amount of *ripA* expressed. A filter disc with 10 µl antibiotic was placed in the center of plate and the diameter of inhibition of growth was measured after 4 days of growth. Antibiotic concentration on disc: carbenicillin (100 mg/ml) and cycloserine (100 mg/ml).

Because depletion of *ripA* may have a marked effect on cell wall structure, we reasoned that strains with diminished expression of *ripA* might have altered susceptibility to antibiotics that target the cell wall. To test this, we grew the *M. Smegmatis* regulated *ripA* strain in the presence of inducer, then washed and spread on plates containing various amounts of inducer (0, 10, 100 ng/ml Tet). A sterilized disc of Whatman paper was placed in the middle of the plate and 10 ul of a single antibiotic was added to the disc. After incubating for 3–4 days, the size of the zone of inhibition was measured (Figure 4). Complete depletion of *ripA* resulted in a remarkably high level of susceptibility to β-lactams (carbenicillin). It is unclear why depletion of *ripA* results in such an increase in susceptibility to β-lactams, which target the transpeptidase reaction required for cross-linking peptidoglycan during cell elongation and division. Susceptibility to cycloserine, an analog of D-alanine that inhibits the formation of the cytoplamsic pentapeptide that is eventually transported across the cell membrane and used to cross-link PG strands, was independent of induction of *ripA*. While increased permeability is often attributed to an observed increase in susceptibility to an antibiotic, depletion of *ripA* did not affect susceptibility to cycloserine, suggesting a more specific defect.

### RipA degrades multiple cell wall substrates

**Figure 5:**
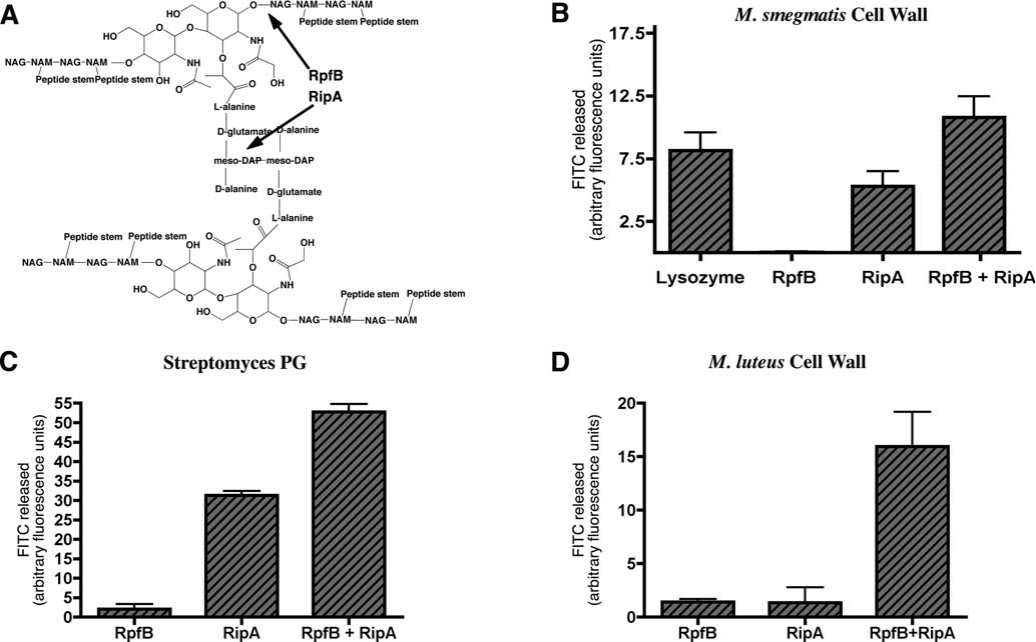
Recombinant RpfB and RipA combine to synergistically hydrolyze peptidoglycan. (A) Diagram of the structure of peptidoglycan, indicating where RpfB and RipA are predicted to have hydrolytic activity. One unit of the peptidoglycan is magnified to show the structure of NAG and NAM as well as the amino acids that are part of the DAP-DAP crosslinkage. Lines connecting NAG to NAM represent β-1,4-glycosidic bonds, while residues connecting NAM to NAM depict peptide cross-linkages. NAG: N-acetylglucosamine, NAM: N-acetylmuramic acid. (B–D) N-terminal GST fusion proteins were expressed and purified from *E. coli*. Equal molar amounts of individual or combinations of proteins were incubated with insoluble FITC-labeled substrate: *M. smegmatis* cell wall (B), Streptomyces peptidoglycan (C), or *M. luteus* cell wall (D). The extent of hydrolysis was determined by measuring the amount of soluble FITC remaining after centrifugation, and thus released during hydrolysis of the insoluble substrate. GST alone, as well as buffer alone, were used to determine background release of FITC and were subtracted from final values. Data are from representative experiments, each done in triplicate. Data are represented as mean +/−SEM.

RipA has been predicted and shown to degrade *M. luteus* cell wall material [25],[26]. To test if RipA hydrolyzes peptidoglycan and cell wall material from other species of bacteria, we determined the enzymatic activity of RipA using a variety of FITC-labeled, cell wall–derived substrates. We expressed RipA as a fusion protein with GST in *E. coli* and purified the fusion protein using affinity chromatography. We found that GST-RipA, but not GST alone, was able to hydrolyze cell wall derived from *M. smegmatis* as well as peptidoglycan purified from *Streptomyces*, and had minimal activity against *M. luteus* cell wall (Figure 5B–D). Therefore, RipA is capable of hydrolyzing cell wall material from several bacterial species.

### The interaction of RipA and RpfB results in synergistic hydrolysis of peptidoglycan

The predicted activity of RipA is to cleave the peptide cross-linkages in peptidoglycan and is distinct from Rpf, which is predicted to cleave glycosidic bonds in peptidoglycan (Figure 5A). Given the close proximity of these predicted cleavage sites, we hypothesized that the interaction of RpfB with RipA may result in enhanced hydrolytic activity. To test this possibility we expressed a portion of RpfB as a fusion protein with GST in *E. coli* and purified it using affinity chromatography.

Using the same assays described above, we found that GST-RpfB alone had minimal ability to degrade cell wall extracts or purified peptidoglycan. However, when GST-RpfB was combined with GST-RipA, activity was more than the sum of individual enzyme activities. The same result was found with all substrates tested (Figure 5B–D). No increase was detected when GST was combined with GST-RpfB or GST-RipA (data not shown). Addition of twice as much Rpf yielded no increase in hydrolysis, while twice as much RipA yielded twice the hydrolysis (data not shown) indicating the assay is in the linear range.

## Discussion

In this work, we demonstrate that RipA is essential for normal cell division in *M. smegmatis*, with its depletion resulting in long, branched filaments and increased susceptibility to a specific cell wall targeting antibiotic. Furthermore, RipA cleaves peptidoglycan and synergizes with RpfB. Taken together, these data support a model where RipA is 1) required for the final stage of cell division, where daughter cells are separated and 2) has peptidoglycan hydrolytic activity that may be modulated by RpfB under certain conditions.

It is unusual that RipA is essential for normal cell division in *M. smegmatis* and, apparently, *M. tuberculosis* [22]. Because bacteria encode a number of hydrolytic enzymes that are, at least in part, functionally redundant, strains carrying deletions of single hydrolase genes are generally viable, though combinations of mutations can result in lack of viability [13]. In *M. smegmatis* and *M. tuberculosis*, *ripA* does not appear to be redundant. Conversely, while *M. marinum* strains carrying mutations in the homologous gene, *iipA*, do have abnormal morphology, they are still able to divide. Mutations in the hydrolytic domain of IipA abolished complementation of the defect, confirming the importance of the hydrolytic activity of IipA [27]. In *M. marinum*, different *rip* paralogues might be able to complement for loss of *iipA*.

None of the *rpf* genes appears to be essential in *M. tuberculosis* and combinations of at least three *rpf* genes can be deleted in *M. tuberculosis* strains while still maintaining normal in vitro vegetative growth [2]. We demonstrate that RpfB is also not essential in *M. smegmatis*. It logically follows that the interaction between RpfB and RipA must not be essential for RipA function during vegetative growth. Of course, it is possible that another Rpf protein is able to compensate for the absence of RpfB, resulting in increased RipA-dependent activity. For example, RpfE is able to interact with RipA [16]. It is also possible that the RipA-RpfB interaction, and subsequent enhanced hydrolytic activity, is required only under special circumstances, such as growth under specific conditions of stress. As noted, RpfB is required for resuscitation of *M. tuberculosis* in a reactivation mouse model [3]. Likewise, deletion of several combinations of three *rpf* genes results in viable bacteria that are unable to resuscitate from in vitro and in vivo resuscitation assays [2]. Thus, the RipA-RpfB interaction may be necessary under certain conditions.

There are several models that might explain the cooperativity seen between RipA and RpfB. One protein might allosterically activate the other, resulting in increased peptidoglycan degradation. Alternatively, both proteins might be fully active, but their association might bring their active sites in close proximity, thus producing cleavage of bonds located near to one another in the peptidoglycan. Since peptidoglycan is a highly cross-linked polymer, nearby cleavages are more likely to effectively degrade peptidoglycan and release fragments.

Several of the most effective antibiotics, including many important antimycobacterial agents, target cell wall synthesis. RipA appears to represent a particular vulnerability for *M. tuberculosis*. In addition to its possible role in reactivation through interaction with Rpf, RipA is essential for normal cell division and is accessible to drugs, given its external localization. Inhibiting the enzymatic activity should block the ability of daughter cells to separate from one another, while blocking protein-protein interactions could result in dysregulation of activity. Thus, RipA is an attractive target for antimycobacterial drug development.

## Materials and Methods

### Strains and culture conditions

*E. coli* XL-1 (Stratagene) strains were used for cloning and *E. coli* BL21 (DE3) (Stratagene) was used for expression of recombinant proteins from the pET41a (Novagen) or pMal (NEB). *Mycobacterium smegmatis* (mc^2^155) and *Mycobacterium tuberculosis* (H37Rv) strains were grown at 37°C in Middlebrook 7H9 broth supplemented with ADC and Tween80 and antibiotic when appropriate.

### Recombinant protein production

The *E. coli* expression strain, BL21(DE3) was used to synthesize each protein following the Novagen manual protocol. Protein concentrations were measured using the Bradford assay, normalized, and confirmed by coomassie-stained polyacrylamide gels.

### Western blotting

Protein samples were combined with 4× Laemmli's SDS PAGE buffer and boiled at 100°C for 10 minutes. Proteins were separated on 10% Tris-tricine polyacrylamide gels by electrophoresis, transferred to nitrocellulose, and probed with specific antibodies using standard techniques.

### Preparation and FITC-labeling of cell wall material

*M. smegmatis* cell wall was prepared as previously described [14]. *Streptomyces* peptidoglycan and lyophilized *M. luteus* cell wall were both obtained from Sigma. The fluorescein isothiocyanate (FITC)-labeled bacterial cell wall was prepared by covalently linking FITC to amine groups in the cell wall. 10 mg FITC (Molecular Probes) was used to label 10 mg of insoluble peptidoglycan or cell wall material following the protocol from Molecular Probes.

### Enzyme assay

Recombinant *M. tuberculosis* proteins were incubated with several FITC-labeled cell wall substrates and assayed for activity by measuring FITC release. 25 µg of Rpf or RipA alone or 25 µg of Rpf and 25 µg RipA combined, were added to 25 µl of 2 mg/ml substrate and 25 µl 4× reaction buffer (50 mM Tris, 10 mM MgCl, 50 mM KCl, 2 mM MnCl, 0.01% Chaps, 100 mM KH_2_PO_4_, pH 5.75). The final volume was brought to 100 µl with H_2_O. As a control, 50 µg of lysozyme was added to *M. smegmatis* cell wall. Similar combinations with GST were also tested. GST alone, as well as buffer alone, were used to determine background release of FITC. After incubating at 30°C with enzyme and buffer for 3–5 days, the insoluble substrate was centrifuged (18,000×g) and soluble FITC was measured with filters for excitation 485 nm and emission 538 nm.

### Generation of depletion strains

Depletion strains were generated as previously described [28]. Briefly, *M. smegmatis*, with the tetracycline repressor gene integrated into the *attB* site, was transformed with a suicide vector containing the first 600 nucleotides of *M. smegmatis ripA* gene under control of the tetracycline operator/promoter system (Ptet). Transformants were selected for hygromycin resistance. Appropriate recombination was confirmed using forward primers to Ptet and Prip (native *ripA* promoter) paired with a reverse primer to the 3′ end of *ripA*. The presence of a product of appropriate size for the former and lacking in the latter, confirmed the desired strain. Attempts to disrupt the *ripA* gene in *M. smegmatis* using a nonreplicating suicide vector designed to recombine into the middle of the gene were unsuccessful (though control knockouts, such as *rpfB*, were successful).

### Depletion strain growth and complementation

The *ripA* and *rpfB* depletion strains were initially grown in 7H9 media containing kanamycin (selecting for TetR) and hygromycin (selecting for inserted pTet) as well as anhydrotetracycline (Tet). Once cultures reached late log-phase or stationary phase, they were centrifuged (2500×g for 5 minutes), washed once with PBS, and resuspended in media with varying amounts of Tet. To test recovery of *ripA* depleted cells, Tet was either added directly to cultures grown without Tet or to fresh media inoculated with cells depleted of *ripA.* To test complementation, the *ripA* gene and its native promoter from *M. tuberculosis* was amplified and cloned into an episomal plasmid containing the *zeocin* gene as a marker. This construct, or the isogenic empty vector, was transformed into the *ripA* depletion strain of *M. smegmatis*. Strains were grown in the presence of tetracycline inducer, washed and inoculated into media lacking inducer. Cultures were monitored by OD_600_ and microscopy. To confirm the essentiality of *ripA*, depletion strains of *M. smegmatis* were grown on a plate with a gradient of inducer generated by placing 10 µl of 10 ng/ml Tet on a paper disc in the center of the plate, resulting in a concentration of inducer highest at the middle of the plate and lowest at the edges.

### Susceptibility to antibiotics assay

The *ripA* depletion strain of *M. smegmatis* was spread on LB agar plates containing different amounts of anhydrotetracycline inducer (ng/ml concentrations) to regulate the amount of *ripA* expressed. A filter disc with 10 µl of carbenicillin (100 mg/ml), isoniazid (10 mg/ml), or cycloserine (100 mg/ml) was placed in the center of plate and the diameter of inhibition of growth was measured after 4 days of growth.

### Microscopy and imaging

*M. smegmatis* strains were centrifuged at 2500×g for 2 minutes, washed with 1ml PBS, and resuspended in 20 µl of PBS containing 50 nM TMA-DPH or 5 µM SYTO 9 for staining membranes or DNA, respectively. Samples were imaged with a Nikon TE-200 100× (NA 1.4) objective and captured with an Orca-II ER cooled CCD camera (Hamamatsu). Final images were prepared using Adobe Photoshop 7.0.
